# Genetic ablation of interacting with Spt6 (*Iws1*) causes early embryonic lethality

**DOI:** 10.1371/journal.pone.0201030

**Published:** 2018-09-12

**Authors:** Arturo Orlacchio, Aaron E. Stark, Claudia Foray, Foued Amari, Tyler Sheetz, Erika Reese, Anna Tessari, Krista La Perle, Dario Palmieri, Philip N. Tsichlis, Vincenzo Coppola

**Affiliations:** 1 Department of Cancer Biology and Genetics, College of Medicine, The Ohio State University, Columbus, Ohio, United States of America; 2 Arthur G. James Comprehensive Cancer Center, The Ohio State University, Columbus, Ohio, United States of America; 3 Genetically Engineered Mouse Modeling Core, The Ohio State University, Columbus, Ohio, United States of America; 4 Department of Veterinary Biosciences, College of Veterinary Medicine, The Ohio State University, Columbus, Ohio, United States of America; 5 Comparative Pathology & Mouse Phenotyping Shared Resource, Columbus, Ohio, United States of America; 6 Molecular Oncology Research Institute, Tufts Medical School, Boston, MA, United States of America; Macau University of Science and Technology, MACAO

## Abstract

IWS1 is an RNA-polymerase II (RNAPII)-associated transcription elongation factor whose biological functions are poorly characterized. To shed some light on the function of this protein at the organismal level, we performed a systematic tissue analysis of its expression and generated *Iws1*-deficient mice. A thorough immunohistochemical characterization shows that IWS1 protein is present in the nucleus of all cells in most of the examined tissues, with few notable exceptions. We also report that ablation of *Iws1* consistently causes lethality at the pre-implantation stage with high expression of the gene in fertilized oocytes. In summary, we are providing evidence that *Iws1* is expressed in all adult organs and it is an essential gene for mouse embryonic development.

## Introduction

IWS1 (interacts with Spt6) is a transcription elongation factor, which was originally identified in the yeast *Saccharomyces cerevisiae* either as a protein interacting with the histone chaperone Suppressor of Ty6 (Spt6, aka SPTH6)[1] or as a suppressor of TATA binding protein (TBP) mutations that impair post-recruitment transcriptional activation. Because of this property, Iws1 in yeast is also known as **S**uppressor of **P**ost-recruitment functions gene **N**umber 1 (*SPN1*)[2].

The full length human IWS1 is an 819 amino acid protein, which contains a C-terminal region that is similar to domain I of the transcription elongation factor TFIIS, and to related domains in Elongin A and the Mediator Complex subunit 26 (Med26). This domain is required for transcriptional regulation and RNA processing. On the other hand, the N-terminal domain of IWS1 has features of the MDN1 (Midasin) domain superfamily, which appears to have a role in ribosomal biogenesis [3].

IWS1’s biological functions are poorly characterized and most of what it is known comes from its association with SPT6. The crystal structure of the SPT6/IWS1 complex has been determined for the SPT6 and IWS1 proteins of *Encephalitozoon cuniculi* [4]. This structure revealed that IWS1 interacts with SPT6 via a bipartite motif within its TFIIS domain, which appears to play an important role in the assembly of transcription complexes. SPT6 interacts with IWS1 via its N-terminal domain, which is highly acidic and unstructured and also binds to nucleosomes [5, 6]. SPT6 interactions with IWS1 and the nucleosomes are mutually exclusive. As a result, IWS1 blocks the formation of the SPT6 nucleosome complex [6]. The N-terminal domain of SPT6 is required for survival, as evidenced by the fact that its deletion gives rise to a lethality phenotype in yeast [7].

SPT6 extensively colocalizes with RNAPII and is crucial for transcription elongation at many, but not all, genes [8–10]. It binds the Ser-2-phosphorylated C-terminal domain (CTD) of RBP1, the large subunit of RNAPII. The SPT6-bound IWS1 functions as an adaptor protein for the assembly of a transcriptional elongation complex that includes the nuclear export factor ALY/REF and the lysine methyltransferase SETD2. In the absence of IWS1 phosphorylation, histone H3 is hypomethylated in transcriptionally active genes and this results in alternative splicing defects [11].

In addition to TBP, Spt6, ALY/REF and SETD2, IWS1 is known to also interact with SPT4, SPT5 [12], and the arginine methyltransferase PRMT5 [13]. The interaction of IWS1 with TBP has been shown to regulate transcriptional initiation [14]. By binding to SPT6, IWS1 is removed from TBP, which can now interact with the SWI/SNF chromatin remodeling complex and initiate transcription [6]. The biological significance of the interaction of IWS1 with SPT4, SPT5 and PRMT5 is not well understood. However, it is known that PRMT5 methylates SPT5 at R700. Moreover, PRMT5 and SPT5 function in concert to regulate transcription, suggesting a potential involvement of IWS1 to PRMT5/SPT5-dependent transcriptional regulation [15].

The gene encoding for the IWS1 human homolog (*hIWS1*) is located on chromosome 2q14.3 and has been shown to be essential for cell proliferation *in vitro* [13].

Mouse *Iws1* is encoded on chromosome 18 the locus produces a 766 amino acid protein. Mouse IWS1 exhibits extensive sequence homology and shares domains with the human homolog while showing the main differences in the N-terminal MDN1 domain.

IWS1 studies in the whole mammalian organism are lacking. In this report we examined the role of IWS1 at the organismal level in mice. Our findings show that IWS1 is widely expressed in adult tissues and that it is localized in the nucleus in a diffuse, or speckled pattern, depending on the tissue and the cell type within a tissue. More important, although in some cell types IWS1 expression in high, in other cell types it is non-detectable. Importantly, the homozygous deletion of IWS1 gave rise to an embryonic lethal phenotype, with the death of the embryos occurring at the preimplantation stage at which the expression of IWS1 is high. The mechanism of embryonic death in IWS1 null embryos remains to be determined.

## Material and methods

### Ethics statement

"Work reported in this manuscript was approved by the IACUC of the Ohio State University. IACUC protocol number 2017A00000067; P.I.: Vincenzo Coppola; Date of Approval: 07/31/2017; Date of Expiration: 07/31/2020."

### Immunohistochemical (IHC) analysis of Iws1 expression in mouse adult tissues

Adult mouse tissues were sectioned and stained at the Comparative Pathology & Mouse Phenotyping Shared Resource of the Ohio State University Comprehensive Cancer Center as previously described [16]. Briefly, tissues were fixed in 10% (v/v) formalin, routinely process, and embedded in paraffin for immunohistochemical characterization.

Formalin-fixed, paraffin-embedded tissues were cut into 4-μm-thick sections, and were dewaxed in xylene, and rehydrated through graded ethanol solutions. Antigens were retrieved by heating the tissue sections at 100°C for 30 min in citrate solution (10 mmol/L, pH 6.0). Then sections were cooled and immersed in methanol in the presence of 0.3% hydrogen peroxide for 15 min to block the endogenous peroxidase activity, subsequently rinsed in PBS for 5 min, and then incubated with the rabbit polyclonal antibody used in the “Human Protein Atlas Project” (Sigma HPA035719) at 4°C overnight. As negative control, slides were incubated with rabbit IgG instead of the primary antibody. The sections were then incubated with horseradish peroxidase-labeled goat against rabbit secondary antibody; Nova Red was used as the chromogen and hematoxylin as the nuclear counterstain. Sections were then dehydrated, cleared and mounted permanently with glass coverslip. Slides were photographed with an Olympus BX45 light microscope with attached DP27 digital camera and corresponding CellSen software (B&B Microscopes Limited, Pittsburgh, PA).

### WB analysis of Iws1 expression

Mouse tissues were homogenized on ice in NP-40 buffer supplemented with Halt Protease and Phosphatase Inhibitor cocktail (Thermo Fisher Scientific). Protein concentration was determined using the Bio-Rad protein assay dye (Bio-Rad). Western blot analysis was performed using 30 to 50 μg proteins run on Mini-PROTEAN TGX precast gels (Biorad).

IWS1 primary antibody was purchased from Cell Signal Technology and used at a dilution of 1:1,000 in 5% BSA in TBS-T. Signals were detected with HRP-conjugated secondary antibodies (GE Healthcare) and the chemiluminescence substrate Supersignal weat pico PLUS (Thermo Fisher Scientific). Equivalent loading among samples was confirmed with anti-GAPDH (cell signal) or with anti-Vinculin (Abcam)

### RT-PCR analysis of Iws1 expression

Total RNA was extracted using TRIzol reagent (Thermo Fisher, Life Technologies) following the manufacturer’s instructions. The quality of RNA was evaluated by the agarose gel electrophoresis and absorbance measurement at 260/280nm. Total RNA (500ng) was reverse transcribed (RT) using the High capacity cDNA reverse transcription kit (Applied Biosystems). Real-time PCR was performed with TaqMan fast advanced master mix (Applied Biosystems). Cycling conditions were set according to manufacturer specifications. Samples were amplified simultaneously in triplicates in one-assay run. Analysis was performed by Prism 7.0 software (Graphpad software) using the Δ-ct method (Applied Biosystems). The TaqMan probes used were Mm00723292_m1 for Iws1 and Mm99999915_g1 for GAPDH.

### Generation of conditional Iws1 knockout mice

The strategy employed for the generation of this model is shown in **S1 Fig**. Briefly, the targeting vector was acquired from the International Knockout Mouse Consortium (IKMC) (vector PG00131_Z_1_C06 of the KOMP-CSD project ID# 44132) and it was electroporated into C57Bl/6-Sv129 hybrid mouse Embryonic Stem (ES) cells. Correctly recombined clones were identified by Southern blotting. Two different clones were used to generate male chimeras, which were subsequently mated to C57BL/6N (Taconic Biosciences) females to obtain F1 offspring positive for the *Iws1*^*tm1a(KOMP)Wtsi*^ allele. In the targeted allele (tm**1a** configuration), Iws1 exon 4 is flanked by two LoxP sites allowing its removal by Cre-mediated recombination, which results in a frame shift of the coding sequence and predicted non-sense mediated decay of the short RNA transcript from the mutated locus (tm**1b** or tm**1d**) (IKMC project 44132).

The primers used for genotyping were:

Primer 1–5’ TACGATGCGCCCATCTACAC

Primer 2–5’ TACCCGTAGGTAGTCACGCA

Primer 3–5’ GCTACCATTACCATTGGTCTGGTGTC

Primer 4–5’ TCAGCAGGCTGTAACAACC

Primer 5–5’ GACATGGCGCAACGCAATTAATG

### Southern blot analysis

Southern blot was performed as previously described [17]. For ES clone screening purposes, we employed a 577 bp IWS1 genomic specific probe external to the targeting vector at the 3’ end of the targeted region (3’ probe) and a LacZ specific internal to the vector probe of 730 bp. Using the 3’ probe and digesting genomic DNA with BglI, a ~20.2 kb fragment the wild type allele and one of 11.4 kb of the mutant allele are observed. Using the LacZ probe only a ~9 kb IWS1 mutant fragment is evident after digestion with BglII demonstrating the presence of the neo/lacZ vector specific cassette.

## Results

### Iws1 expression is ubiquitous in mice

Understanding the temporal and spatial patterns of *Iws1* expression during development and in adult tissues was the first step in our effort to understand the biological function of IWS1 at the organismal level.

Information based on microarray studies and RNA-Seq analyses currently available from public databases suggests that *Iws1* is widely expressed (www.proteinatlas.org). However, since mRNA and protein levels might not necessarily correlate [18–20], we decided to focus on determining the IWS1 expression at the protein level. A preliminary Western Blot (WB) analysis revealed that IWS1 was present in all examined tissues (not shown). In order to clarify whether all the cell types were positive for IWS1, we decided to perform a systematic immunohistochemical (IHC) survey using a specific validated antibody. To this aim, we employed the rabbit polyclonal antibody used in the “Human Protein Atlas Project” (http://www.proteinatlas.org/ENSG00000163166/antibody), which was raised using peptides conserved in human and mouse.

**Fig 1:**
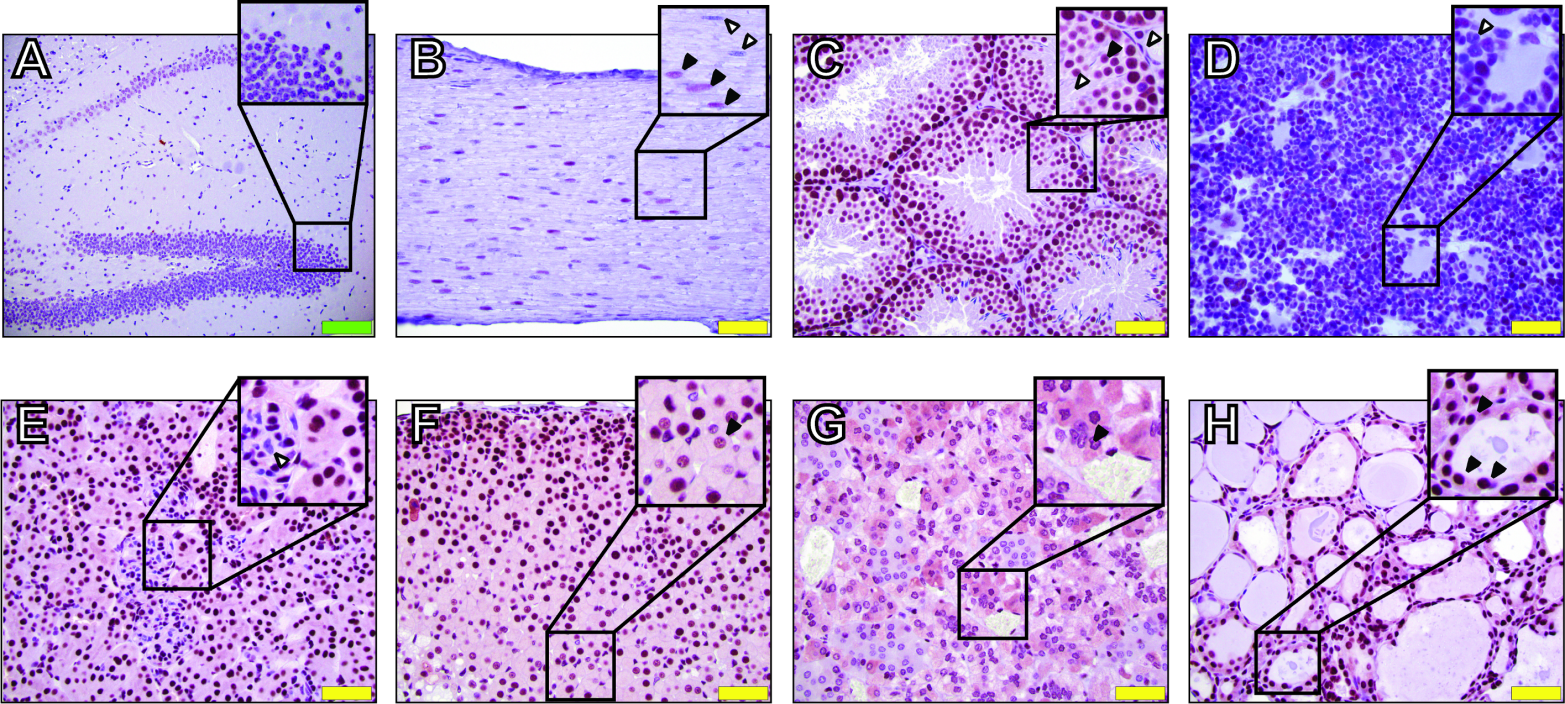
Immunohistochemical analysis of IWS1 expression in mouse tissues. A) Hippocampus, inset shows negative cells. B) Sciatic nerve, inset shows IWS1 negative (white arrows) and positive (black arrows) nuclei. C) Testis, inset shows IWS1 negative Leydig cells and mature sperm (white arrows) and positive Sertoli cells (black arrows). D) Bone marrow, inset shows IWS1 negative segmented neutrophils (white arrow). E) Renal cortex, inset shows Iws1-negative renal glomerular mesangial cells. F) Adrenal cortex, inset shows speckled nuclear staining for IWS1 (black arrows). G) Adrenal medulla, inset shows IWS1 cytoplasmic staining in chromaffin cells (black arrows). H) Thyroid, inset shows IWS1 cytoplasmic staining in follicular epithelial (cuboidal) cells. Green and yellow scale bars indicate 100 **μ**m and 50 **μ**m, respectively.

We stained a comprehensive set of tissues representing 10 different systems, including different areas of the brain and nervous system (**Fig 1**, **S1 Fig**). In general, IWS1 protein is clearly expressed in the nucleus of most cell lineages and tissues examined. However, we also noticed that IWS staining is below detectable levels in some of the cells of selected tissues (**Fig 1**). Specifically, neurons of the hippocampus and some of the sciatic nerve cells appear not to express IWS1 (**Fig 1A**, **Fig 1B,** inset). In the testis, spermatozoa and Leydig cells IWS1 staining was below detectable levels, while it was evident in Sertoli cells (**Fig 1C**). Additional apparent IWS1-negative cells include mature segmented neutrophils in the bone marrow (**Fig 1D**) and mesangial cells in the glomeruli of the kidney (**Fig 1E**). In most cells expressing IWS1, the protein was localized primarily in the nucleus and the staining pattern was either “speckled” or uniform (adrenal cortex, **Fig 1F**, inset). Finally, IHC staining of endocrine organs showed a diffuse cytoplasmic positivity in chromaffin cells of the adrenal medulla (**Fig 1F and 1G**) and thyroid follicular epithelial (cuboidal) cells (**Fig 1H**).

Taken together, these results show that IWS1 protein is ubiquitously expressed in cell nuclei of mouse adult tissues. They also demonstrate that specific cell populations in selected tissues do not show detectable levels of IWS1 protein.

### Ablation of Iws1 causes pre-implantation lethality during mouse development

To study the function of IWS1 during mouse development, we generated an *Iws1* conditional knockout mouse model (*Iws1* cKO). To establish whether *Iws1* was essential for mouse development, we proceeded to ablate this gene in the germ line by Cre-mediated recombination. This was done by crossing the heterozygous *Iws1*^+/tm1a^ mice with the B6.FVB-Tg(EIIa-cre)C5379Lmgd/J (JAX stock number 003724) mice. EIIa-Cre completely ablates floxed genes prior to implantation [21] and the cross generates *Iws1*^+/tm1b^ mice (**S2 Fig**). Probing western blots of lysates derived from mouse tissues of heterozygous and wild type (WT) mice revealed that heterozygous mice display about half of the amount of protein present in WT animals, and confirmed that the removal of exon 4 disrupts the expression of Iws1 (**S2 Fig**). Crosses of heterozygous *Iws1*^+/tm1b^ parents produced a total of 320 live pups and none of them was a homozygous mutant (**S1 Table**). These results demonstrate that *Iws1* genetic ablation carries an embryonic lethal phenotype.

**Fig 2:**
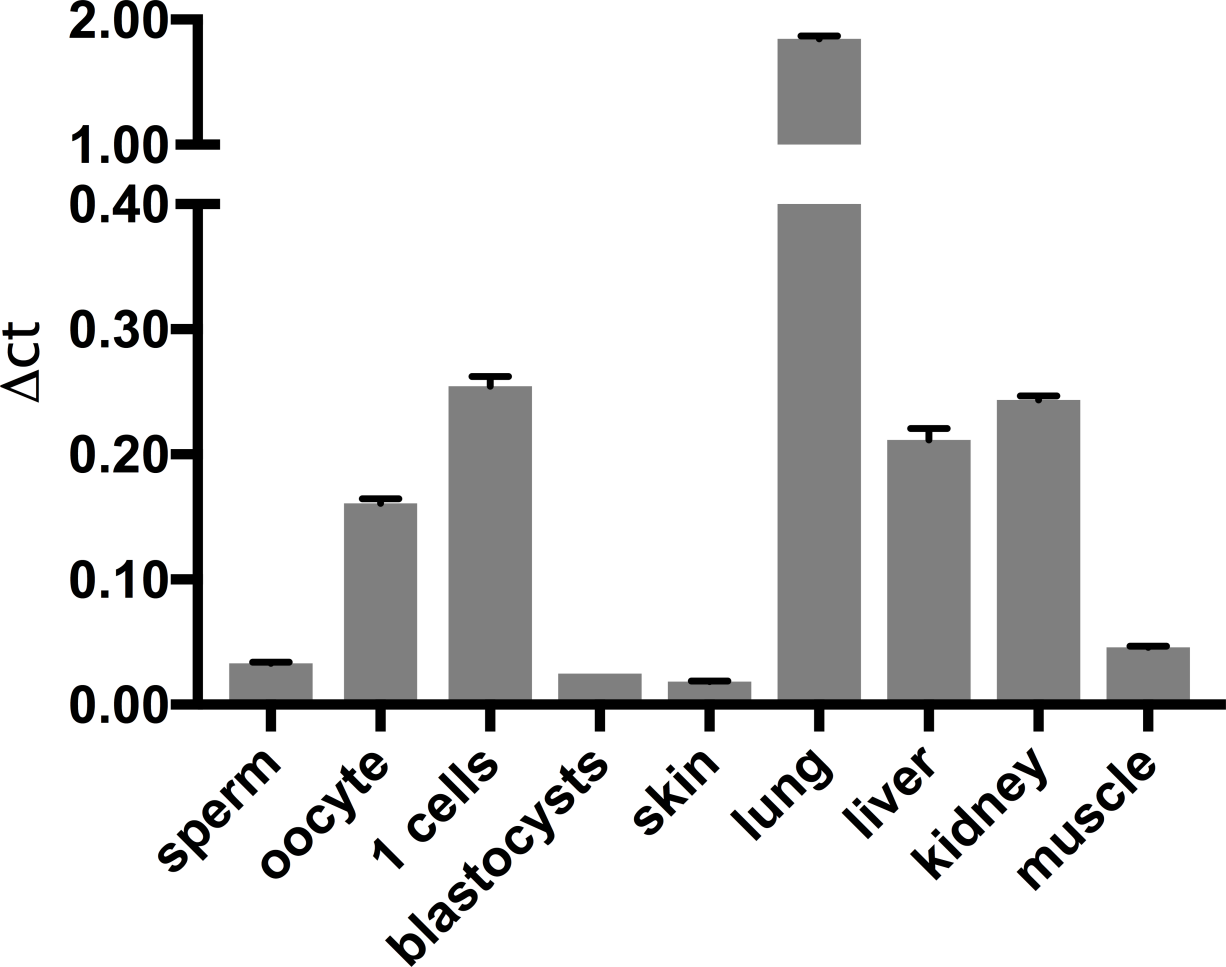
RT-PCR of pre-implantation embryos and other tissues. Iws1 mRNA expression levels in mouse adult tissues, germinal cells and pre-implantation stage embryos. Standard deviation (black bar) is shown for each sample. Samples were amplified simultaneously in triplicates in one-assay run. Experiments were performed three times.

Having established that the complete ablation of *Iws1* results in the absence of live born pups, we proceeded to collect embryos at different stages of post-implantation development. No homozygous live embryos were obtained at any of the analyzed developmental stages. The preceding observations raised the question whether *Iws1* is expressed in mouse embryos prior to implantation. Using quantitative RT-PCR we observed that this is indeed the case. Specifically, we found that *Iws1* mRNA is clearly detectable in fertilized mouse eggs while its expression decreases in blastocysts (E3.5d) (**Fig 2**). The *Iws1* expression pattern in early embryos, combined with our failure to detect *Iws1* null live embryos at later stages of development, suggested that *Iws1* might indeed be essential for embryo development prior to implantation. To test this hypothesis, we set up timed pregnancies and collected blastocysts. Out of a total of 64 viable blastocysts, none was homozygous for the ablation of *Iws1* (**Table 1**).

**Table 1:** Pre-implantation embryos from *Iws1*^*+/tm1b*^ het x het crossing.

*Iws1* Genotype	Number Observed	Number expected	χ^2^ value	p-value
Wild-Type	25	16	5.0625	0.07956
Heterozygous	39	32	1.53125	0.465
Homozygous	0	16	16	3.355e-4

Blastocyst stage embryos were collected and genotyped. Statistical analysis of observed genotype distribution was performed using the chi-square test.

These results confirmed that *Iws1* is expressed at pre-implantation stages and that embryos fail to develop in its absence.

## Discussion

From publicly available data, IWS1 seems to be ubiquitously expressed in mammalian organisms (www.proteinatlas.org). However, a definitive analysis of its expression in mouse adult tissues has not been reported yet. Here, we show that IWS1 protein is ubiquitously expressed. Most of the tissues analyzed by IHC show positive nuclear staining using a specific anti-IWS1 antibody (**Fig 1** and **S1 Fig**). Most of the cells show an intense positivity at the nuclear level; in some case the staining is less intense and more “speckled” (**S1 Fig**). However, few selected cells in specific tissues like hippocampus, sciatic nerve, fully mature segmented neutrophils in the bone marrow and mesangial cells in the renal glomerulus show expression of this transcription factor below detectable levels (**Fig 1**). Interestingly, mature spermatozoa appeared also negative for IWS1 expression (**Fig 1**). Since we used one of the two antibodies employed by the Human protein Atlas (HPA035719), we compared our observations in cell types where we did not detect IWS1 expression with those of the same human cell populations available from the HPA (**S2 Table**). While, HPA035719 shows no signal in the mouse cells that we analyzed, weak staining is reported in the HPA human corresponding collection. However, a different antibody (HPA061866) used for detection of human IWS1 shows medium staining in all the cell types in question (**S2 Table**). This analysis indicates that our results might be affected by limitations inherent to our staining protocol or intrinsic to the HPA035719 antibody in its sensitivity in detecting mouse IWS1. Nevertheless, our observations might also suggest that *Iws1* might not be essential for all cell types. Specific more differentiated stages of maturation in certain cell types might not require the presence IWS1. Further studies will be required to clarify how *Iws1* expression is regulated, as well as what is the biological relevance of the uniform intense and “speckled” patterns of nuclear staining. IWS1 is known to be localized in the nucleus. However, we observed two different endocrine cell types (chromaffin cells of the adrenal medulla and thyroid follicular epithelial cells) demonstrating a diffuse cytoplasmic positivity by IHC. It will be extremely interesting to establish whether IWS1 is actually present outside the nucleus or if this positivity is an artifact due to intrinsic characteristics of those cell lineages. Due to the similarities with Midasin at the N-terminal region [3], it is tempting to speculate that in those cells IWS1 is in the cytoplasm actively participating to ribosomal biogenesis. However, further experiments will be required to prove this hypothesis.

Due to its interactions with RNAPII cofactors, IWS1 seems to be essential in coupling RNA transcription elongation to splicing and nuclear export in conjunction with chromatin modifications acting as signals to regulate RNA processing [9, 10]. Accordingly, a CRISPR/Cas9 high-throughput screening showed that IWS1 is essential for both human cell lines tested [22]. In fact, the human IWS1 homolog was already reported to be essential for cell proliferation *in vitro* [13].

In line with those results, we report here the generation of an *Iws1* complete knockout model and demonstrate that the ablation of this transcription factor causes early embryonic lethality. In fact we were not able to collect any viable homozygous *Iws1* knockout blastocysts (3.5 days of development; **Table 1**). An analysis of *Iws1* mRNA showed that the gene is expressed at higher levels in fertilized oocytes, than in blastocysts and other adult tissues. Therefore, it is likely that the absence of *Iws1* causes death of embryos at the one-cell stage. Interestingly, we also detected a significant amount of *Iws1* mRNA in non-fertilized oocytes (**Fig 2**), indicating that the *Iws1* mRNA may be transmitted directly from mothers to the progeny because it is needed at the very first stages of development. While we did not perform any experiment to study the molecular details of the early embryo lethality, we can speculate that IWS1 expression is absolutely essential during the very first hours post-fertilization. Possibly, in the absence of IWS1 lethal consequences might be a result of the potential inability of SetD2 to bind PolII H_3_K_36_me3 marks and the CTD of RNAPII [10]. Therefore, lack of IWS1 might impair the transcription and the correct splicing of genes that are crucial to initiate the zygote proliferation and differentiation program.

Recently, *IWS1* has been shown to be highly expressed in malignancies [11]. In particular, IWS1 seems to be an attractive therapeutic target for lung, skin and breast cancers. Despite of the lethality of *Iws1* null embryos, our IHC results suggest that specific mammalian cells might be able to survive and proliferate in the absence of IWS1 (**Fig 1**).

The fact that mutations in Spt6 abolishing the interaction between Spt6 and Iws1 are lethal in yeast [7], suggested that IWS1 may be an essential gene, a hypothesis supported by ablation of the yeast Iws1 gene [2]. A CRISPR/Cas9 screen in haploid mammalian cells suggested that IWS1 gene is an essential gene, also in mammals [22]. However, the requirement for IWS1 in mammalian cells may be cell type specific. Earlier studies from the Tsichlis lab had shown that the expression of IWS1 varies widely between normal and tumor cells, with the tumor cells expressing significantly higher levels [11]. The expression also varies between different tumor cell lines. Importantly, a highly efficient knockdown of IWS1, significantly inhibited the rate of proliferation of cell lines expressing high levels of IWS1, but it had no effect on the rate of proliferation of cell lines expressing low IWS1 levels. Therefore, the question of whether the partial and the complete loss of IWS1 may have drastically different phenotypes and whether these phenotypes are cell type specific requires additional studies to be firmly addressed.

Targeting specific post-translational modifications like phosphorylation enacted by Akt of IWS1 S720, which is particularly abundant in cancer [11], could be a potential alternative anti-cancer strategy.

In summary, here we report that IWS1 is expressed in a wide range of mouse cells and that its ablation causes pre-implantation embryonic lethality. Therefore, targeting of IWS1 as a possible therapeutic approach to treat cancer requires caution and further studies.

## Supporting information

S1 Fig Immunohistochemical analysis of IWS1 expression in mouse tissues. *IWS1* IHC staining of multiple mouse tissues:
A-C) Cerebrum (A), Cerebellum (B) and Spinal Cord (C)
D-F) Aorta (D), Heart (E) and Lung (F)
G-I) Small Intestine (G), Large Intestine (H) and Cecum (I)
J-K) Liver (J) and gall bladder (K)
L-M) Salivary Gland (L) and Pancreas (M)
N-Q) Thymus (N), Spleen (O), Lymph Node (P) Peyer’s Patch (Q)
R-U) Ovary (R), Uterus (S), Mammary Gland (T), and Prostate Gland (U)
V-X) Urinary Bladder (V) and Renal Papilla (X)
Y-AA) Skin (Y), Skeletal Muscle (Z) and Brown Adipose Tissue (BAT, AA).
(PDF)

S2 Fig *Iws1* targeting strategy and cKO mouse generation. A) The “knock-out first” vector to target *Iws1* gene on mouse chromosome 18 was acquired from the IKMC. After recombination, correctly targeted clones bear a tm1a configuration in which Exon 4 is flanked by two loxP sites and a cassette containing a strong splice-acceptor sequence (En2 SA) followed by insertion of the LacZ gene in the third intron. Therefore, the cassette can allow tracking of Iws1 expression *in vivo*. The entire cassette (En2 SA-LacZ-neo) can be removed by Flpe recombination to obtain the allele tm1c (Iws1^fl/fl^) that can, in turn, result in a null allele (tm1d; *Iws1*^*null/null*^) after Cre recombination. However, *Iws1* null animals can also be obtained by crossing tm1a animals immediately to ubiquitous Cre transgenic lines resulting in the tm1b configuration, which is still useful for gene expression tracking. Importantly, removal of Exon 4 is predicted to result in a frame shift mutation. B) Example of PCR screening of mouse tail DNA for the presence of the conditional allele with the indicated primers. C) Example of PCR screening of mouse tail DNA for the presence of the tm1b null allele. D) Southern blot analysis of ES clones. Genomic DNA was digested with BglI (when using the 3’ probe) or with BglII (when using the LacZ probe). Size of the expected fragments are shown in panel A. E) Real time PCR analysis of moue lung tissue comparing RNA levels of Iws1 in wild type and tm1b heterozygous mice. F) Representative Western Blot analysis of mouse lung tissue showing that tm1b heterozygous animals have reduced amounts of IWS1 protein.
(EPS)

S1 Table Live pups from *Iws1* het x het crossing. (DOCX)

S2 Table Immunohistochemical staining comparison between mouse (our observations) vs human tissues (Human Protein Atlas). Primary data.
(DOCX)

S1 File NC3Rs ARRIVE Checklist PONE-D-18-10148.pdf. (PDF)

S2 File Primers 1&2 and 5&4. (PPTX)

S3 File Primers 3&4. (PPTX)

S4 File Scan3probe. (JPEG)

S5 File ScanIWS1 Southern LacZ. (TIFF)

S6 File IWS1. (TIFF)

S7 File Vinculin. (TIFF)
