# Supplementary figures and images for: Genetic ablation of interacting with Spt6 (Iws1) causes early embryonic lethality
Source: PLoS One. 2018 Sep 12;13(9):e0201030. doi: 10.1371/journal.pone.0201030 (PMC6135376; doi:10.1371/journal.pone.0201030)

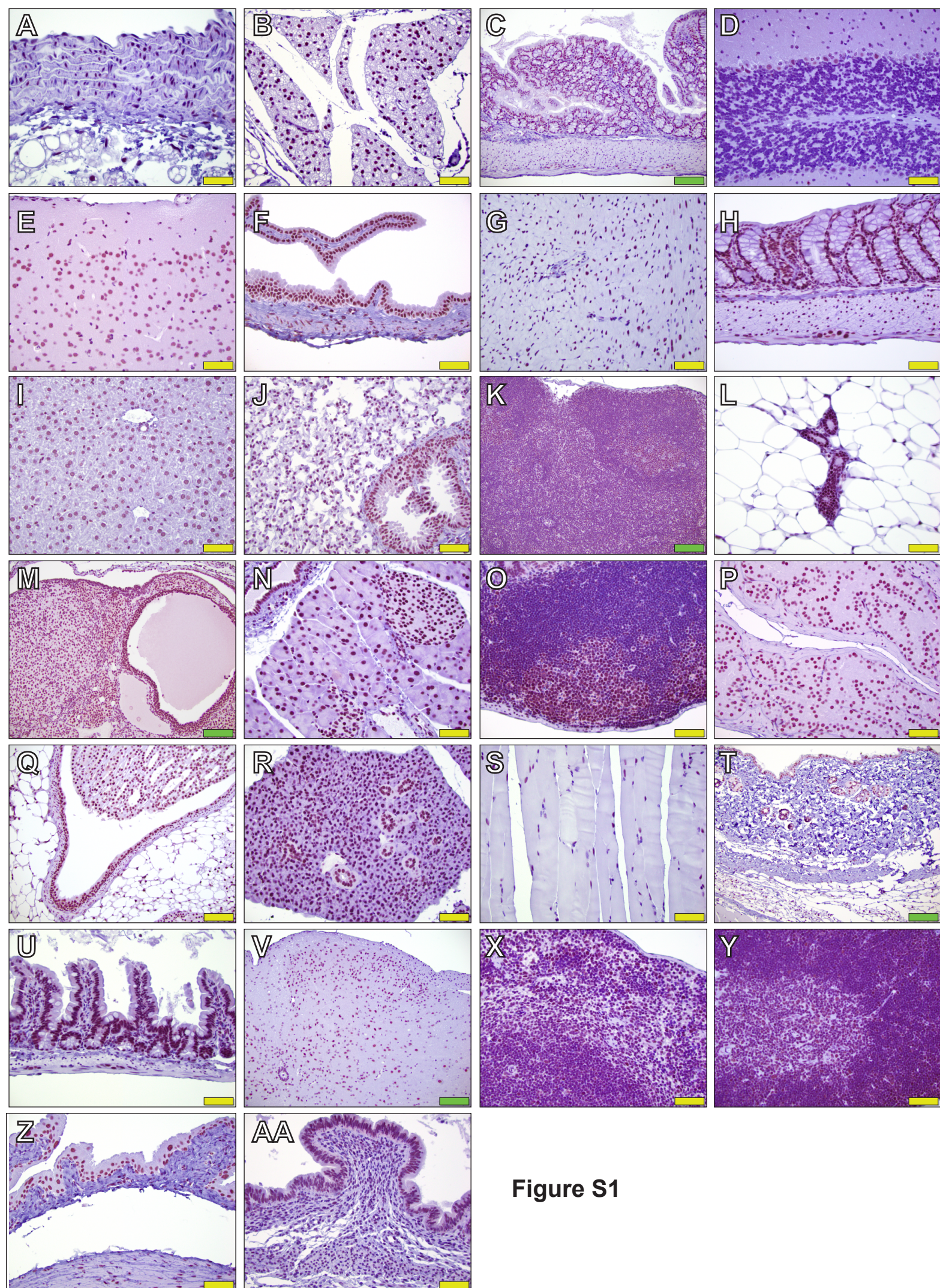

**Figure S1**

Supplement: S1 Fig — IWS1 IHC staining of multiple mouse tissues: A-C) Cerebrum (A), Cerebellum (B) and Spinal Cord (C) D-F) Aorta (D), Heart (E) and Lung (F) G-I) Small Intestine (G), Large Intestine (H) and Cecum (I) J-K) Liver (J) and gall bladder (K) L-M) Salivary Gland (L) and Pancreas (M) N-Q) Thymus (N), Spleen (O), Lymph Node (P) Peyer’s Patch (Q) R-U) Ovary (R), Uterus (S), Mammary Gland (T), and Prostate Gland (U) V-X) Urinary Bladder (V) and Renal Papilla (X) Y-AA) Skin (Y), Skeletal Muscle (Z) and Brown Adipose Tissue (BAT, AA). (PDF) [file pone.0201030.s001.pdf]

## Slide 1
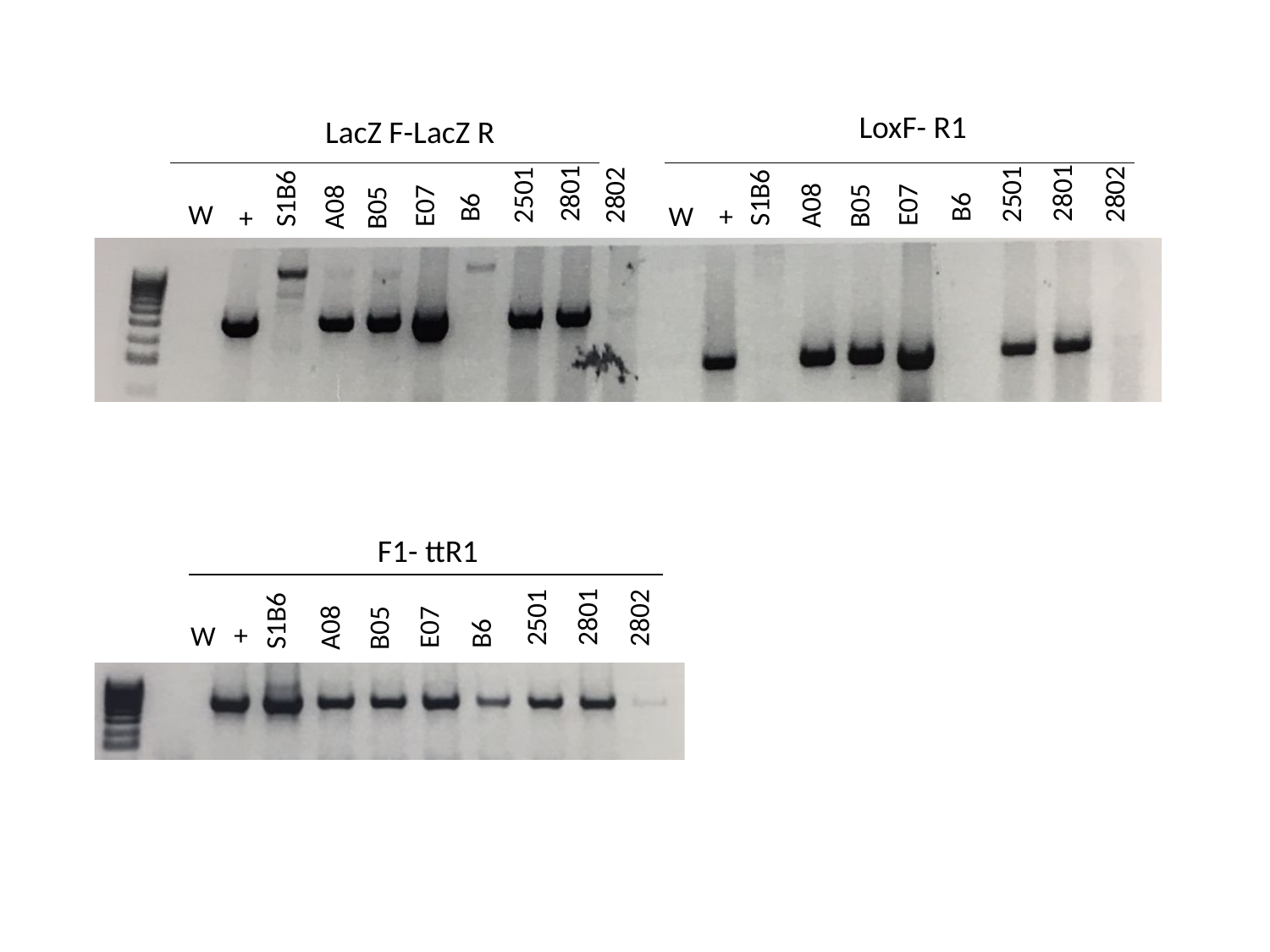

LoxF- R1
LacZ F-LacZ R
2801
2501
2802
S1B6
A08
B05
E07
B6
W
+
2801
2501
2802
S1B6
A08
B05
E07
B6
W
+
F1- ttR1
2801
2501
2802
A08
B05
E07
+
W
S1B6
B6

Supplement: S2 File — (PPTX) [file pone.0201030.s006.pptx]

## Slide 1
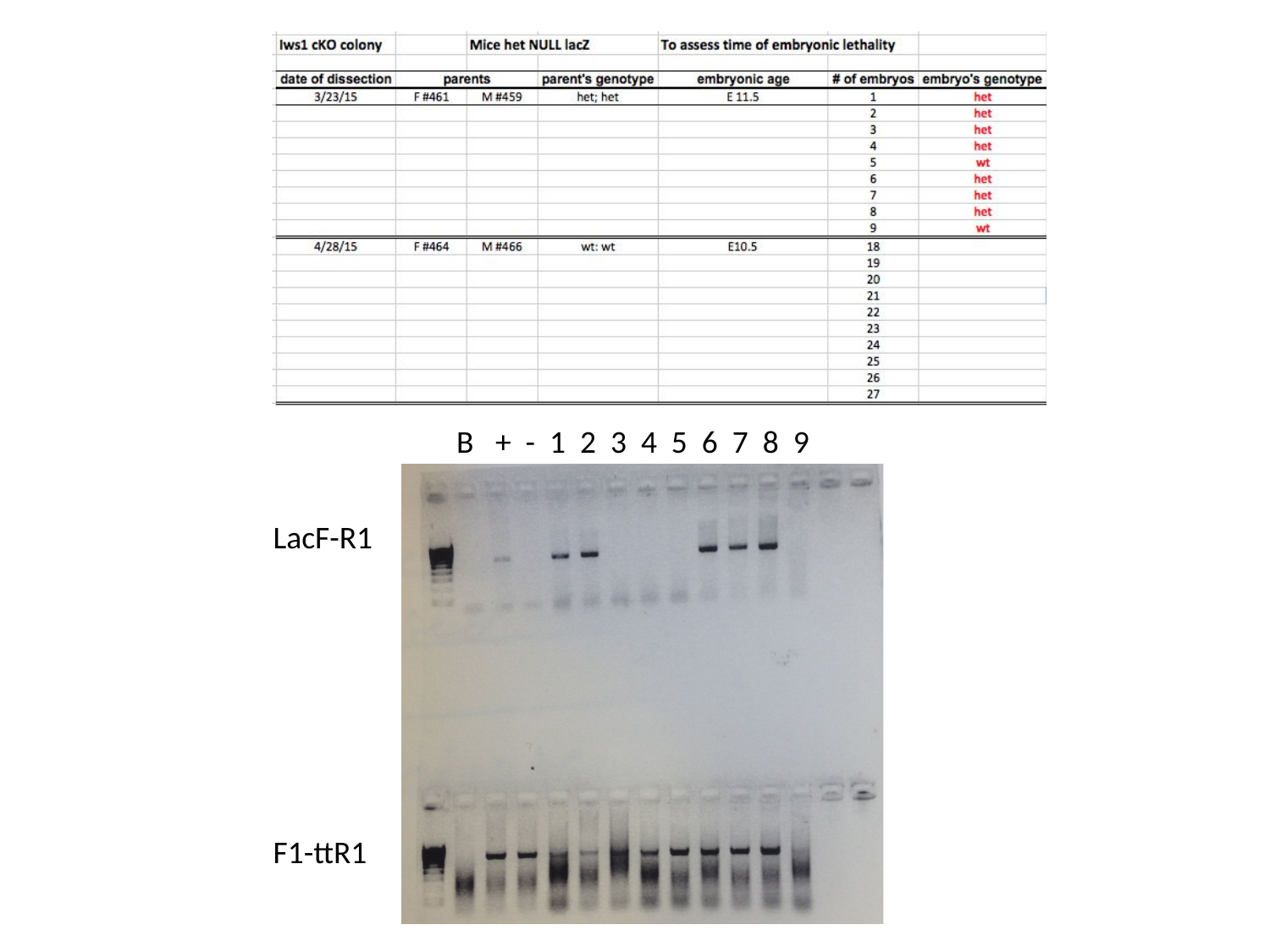

B + - 1 2 3 4 5 6 7 8 9
LacF-R1
F1-ttR1

Supplement: S3 File — (PPTX) [file pone.0201030.s007.pptx]

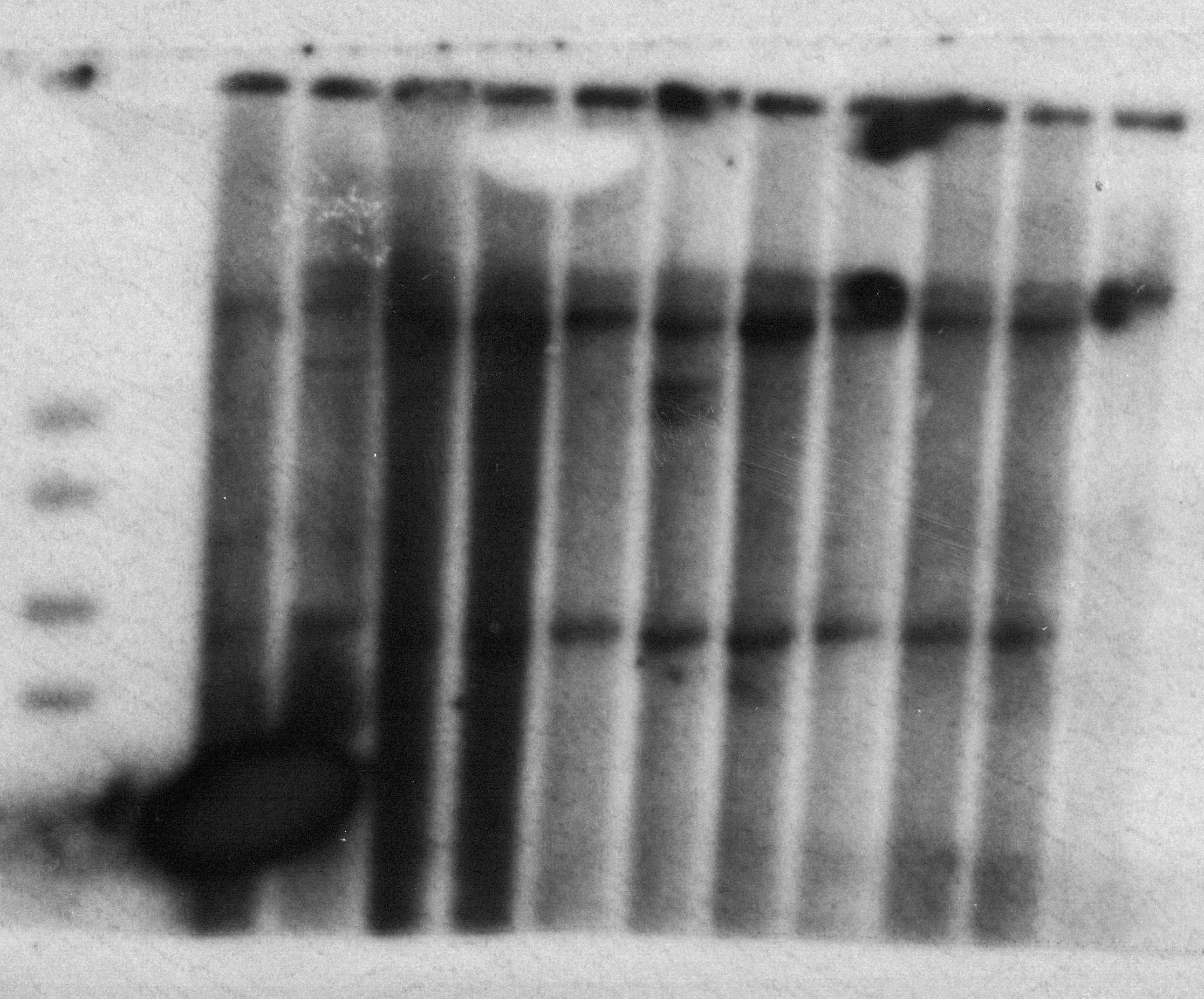

Supplement: S4 File — (JPEG) [file pone.0201030.s008.jpeg]

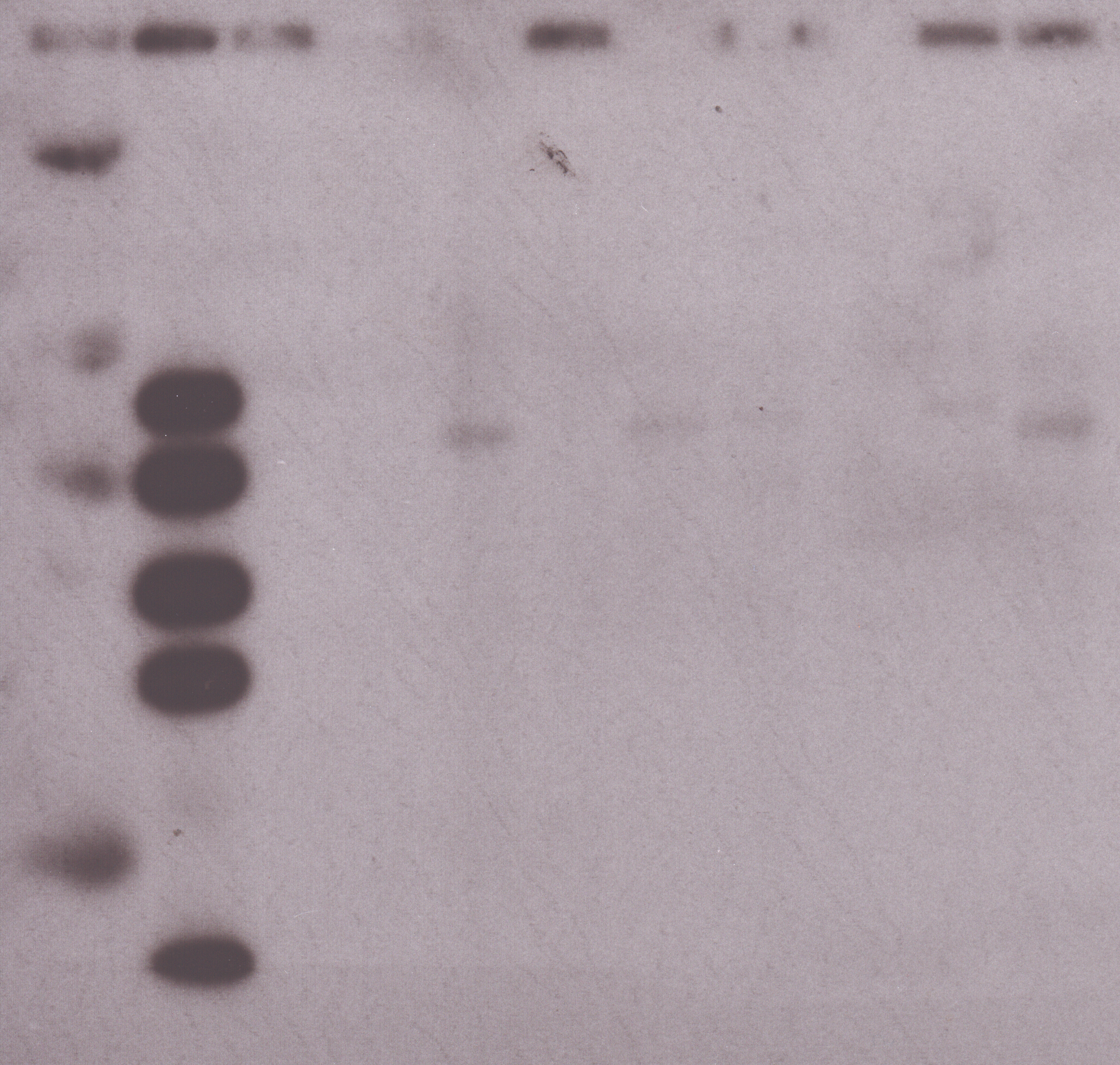

Supplement: S5 File — (TIFF) [file pone.0201030.s009.tiff]

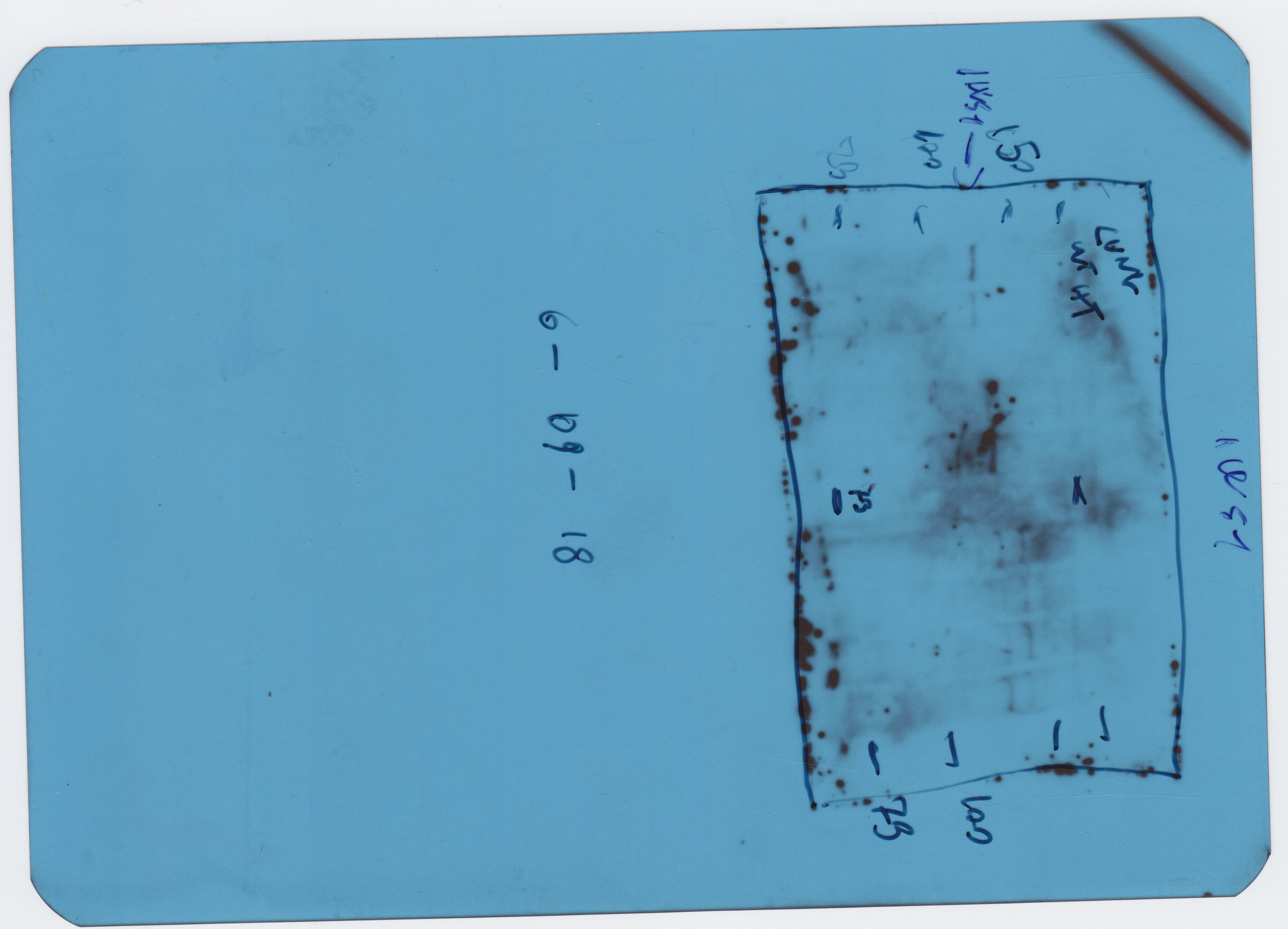

Supplement: S6 File — (TIFF) [file pone.0201030.s010.tiff]

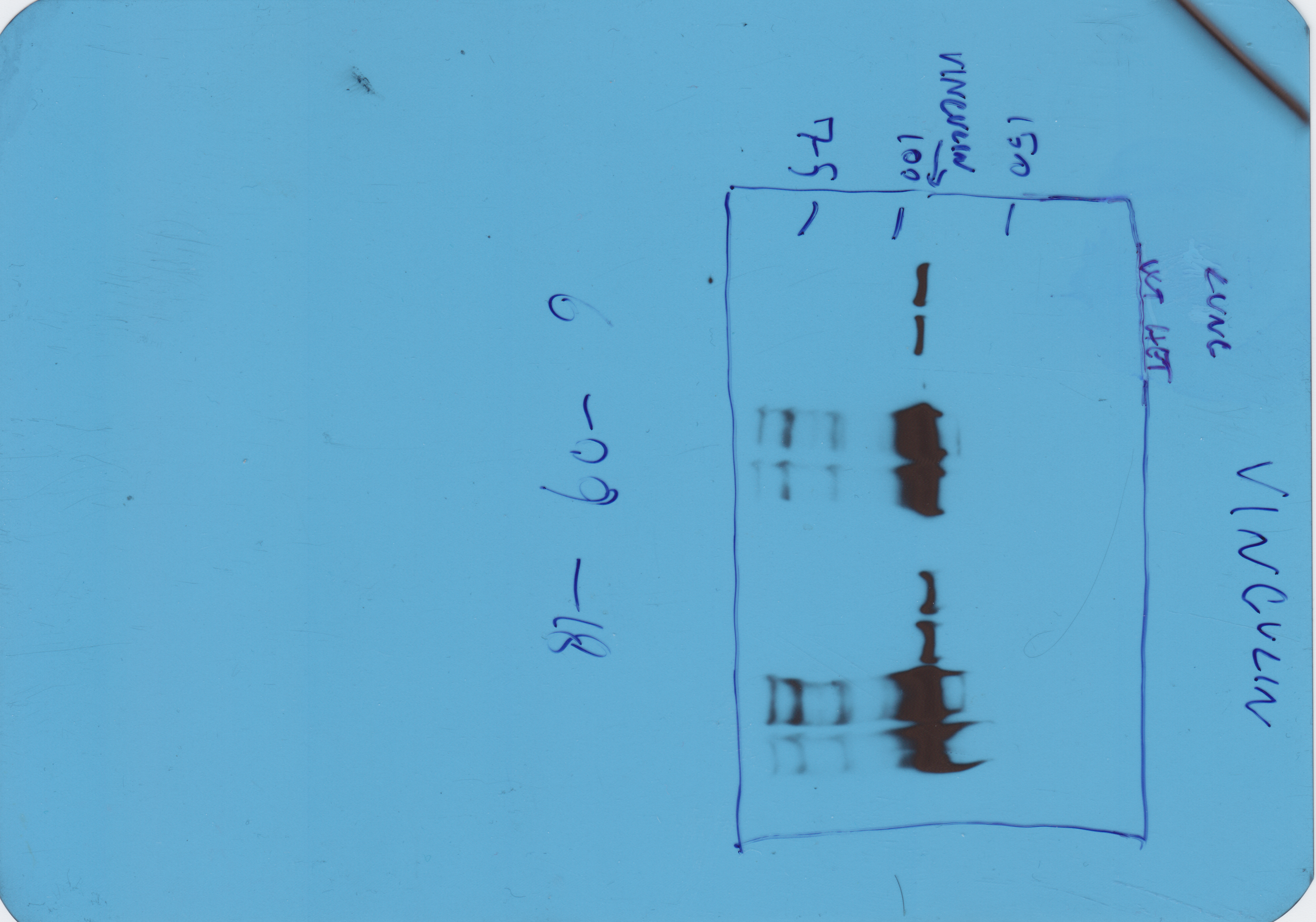

Supplement: S7 File — (TIFF) [file pone.0201030.s011.tiff]
