# Supplementary material for: Genetic ablation of interacting with Spt6 (Iws1) causes early embryonic lethality
Source: PLoS One. 2018 Sep 12;13(9):e0201030. doi: 10.1371/journal.pone.0201030 (PMC6135376; doi:10.1371/journal.pone.0201030)
Supplement: S1 Table — (DOCX) [file pone.0201030.s003.docx]

**S1 Table**: Live pups from *Iws1* het x het crossing.

Statistical analysis of observed genotype distribution for live pups obtained from *Iws1* het x het crossing. Results have been analyzed with chi-square test.

| *Iws1* Mouse Genotype | Number Observed | Number expected | χ^2^ value | p-value |
| --- | --- | --- | --- | --- |
|  |  |  |  |  |
| Wild-Type | 88 | 80 | 0.8 | 0.67032 |
|  |  |  |  |  |
| Heterozygous | 232 | 160 | 32.4 | 9.214e-8 |
|  |  |  |  |  |
| Homozygous | 0 | 80 | 80 | 4.248e-18 |
