# Supplementary material for: Genetic ablation of interacting with Spt6 (Iws1) causes early embryonic lethality
Source: PLoS One. 2018 Sep 12;13(9):e0201030. doi: 10.1371/journal.pone.0201030 (PMC6135376; doi:10.1371/journal.pone.0201030)
Supplement: S2 Table — Primary data. (DOCX) [file pone.0201030.s004.docx]

**S2 Table**: Immunohistochemical staining comparison between mouse (our observations) vs human tissues (Human Protein Atlas).

The staining with two different antibodies is compared. The staining by HPA035719 used in our investigation of IWS1 expression in mouse did not show nuclear staining in the listed tissues only in partial agreement with the staining observed in the HPA collection. Interestingly, HPA035719, the other antibody used for detection of IWS1 in human tissues, is positive (medium) in all the cell types in question.

|  |  |  | Antibody |  |  | Antibody |
| --- | --- | --- | --- | --- | --- | --- |
|  |  |  | HPA035719 |  |  | HPA061866 |
| Cell type |  | Our observations |  | Human Protein Atlas |  | Human Protein Atlas |
|  |  | (mouse) |  | (human) |  | (human) |
|  |  |  |  |  |  |  |
| Hippocampal neurons |  | not detected |  | weak/not detected |  | medium |
|  |  |  |  |  |  |  |
| Kidney glomeruli cells | | not detected |  | not detected |  | medium |
|  |  |  |  |  |  |  |
| Testis Leydig cells |  | not detected |  | medium |  | medium |
|  |  |  |  |  |  |  |
| Thyroid glandular cells | | diffuse  cytoplasmic |  | not detected |  | medium |
